# A simplified two-dimensional culture system supports meiotic progression during mouse spermatogenesis *in vitro*

**DOI:** 10.1371/journal.pone.0342007

**Published:** 2026-02-05

**Authors:** Yuki Yokoi, Yuki Yamashita, Koichi Uemura, Hisakazu Odaka, Kazuhide Makiyama, Takehiko Ogawa, Takuya Sato, Mitsuru Komeya

**Affiliations:** 1 Department of Urology, Yokohama City University School of Medicine, Yokohama, Kanagawa, Japan; 2 Department of Regenerative Medicine, Yokohama City University Graduate School of Medicine, Yokohama, Japan; University of Hyderabad, INDIA

## Abstract

*In vitro* spermatogenesis (IVS) remains a major challenge due to its complexity and extended duration. Although organ culture of neonatal mouse testes can support complete spermatogenesis, its three-dimensional structure limits precise observation and control of culture conditions, resulting in heterogeneous oxygen and nutrient gradients. Two-dimensional (2-D) systems provide greater accessibility but often induce accelerated or abnormal meiosis. In this study, we present a simplified, serum free 2-D culture system that enables meiotic progression of mouse germ cells to the mid pachytene stage. Testicular cells from *Acrosin* (*Acr*)-GFP transgenic mice (2.5 to 10.5 days *postpartum*: dpp) were enzymatically dissociated and cultured in APEL medium, a chemically defined and serum free basal formulation. The medium was supplemented with bovine pituitary extract (BPE), follicle stimulating hormone (FSH), and testosterone (BFT medium). Meiotic progression was monitored in real time by GFP fluorescence and further assessed by immunocytochemistry and nuclear spread analysis. Testicular cells consistently formed Sertoli cell monolayers and supported germ cell differentiation to mid pachytene stage. GFP-positive cells appeared around days 17.5 to 21.5 of culture, reflecting a modest delay compared to organ culture. GFP-positive cells were observed not only in 7.5 to 10.5 dpp cultures, but also in 2.5 to 6.5 dpp testes lacking preexisting spermatocytes, indicating *de novo* induction of meiosis. BFT medium, compared to APEL medium, increased the frequency of GFP-positive wells and the per-well duration of GFP expression, reaching statistical significance in the 9.5–10.5 dpp group (p = 0.021). Nuclear spread analysis confirmed synapsis through pachytene, although cells did not progress to diplotene. This simplified culture system offers a robust and accessible platform for studying early meiotic events and optimizing IVS.

## Introduction

Spermatogenesis is a multistage differentiation process that begins with vigorous mitotic expansion of spermatogonia, proceeds through homologous chromosomal recombination and meiotic reduction to haploidy, involves extensive remodeling of cellular morphology and nuclear chromatin, and culminates in the extrusion of residual cytoplasm, leading to spermiation [1]. This finely orchestrated program has been evolutionarily optimized to maximize reproductive efficiency in each species. Therefore, due to such evolutionary refinement, spermatogenesis follows a precisely regulated sequence that takes more than one month to complete in most mammals. In fact, the full progression from spermatogonial stem cells to mature spermatozoa requires approximately 35 days in mice and 74 days in humans [2]. Owing to the intricacy of this developmental program, the *in vitro* reconstruction of spermatogenesis remained an unmet goal for more than a century. In 2011, however, our group demonstrated that neonatal mouse testis fragments cultured on agarose gel achieved complete IVS, producing functional sperm that successfully generated offspring following microinsemination [3]. This method has since been replicated by many independent research groups, and IVS using mouse testis tissue culture is now becoming a standard procedure [4–8]. Nonetheless, the three-dimensional architecture of tissue cultures limits precise observation and control of culture conditions, resulting in heterogeneous oxygen and nutrient gradients.

To address these limitations, 2-D cell-culture systems have attracted increasing attention. Such systems enable direct microscopic observation, provide a more homogeneous culture environment, and facilitate simultaneous testing of multiple conditions using a single specimen, thereby reducing the number of animals required. A persistent challenge, however, is meiotic arrest at the pachytene stage [9]. To date, only one study has generated functional haploid spermatozoa entirely *in vitro* using a 2-D culture system [10]. In that report, meiotic progression occurred substantially faster than *in vivo*, which may pose challenges in evaluating how closely the *in vitro* differentiation reflects natural developmental processes and timing.

In the present study, we developed a simple 2-D culture system that supports mouse germ cell progression to the mid-pachytene stage with a modest delay relative to *in vivo* timing. We demonstrate progressive proliferation and differentiation of germ cells, provide further evidence that mouse meiosis can proceed *in vitro*, and define both the extent and the limitations of meiotic progression under these conditions.

## Materials and methods

### Animals

Double-transgenic *Acrosin*-GFP/*H3.3*-mCherry (hereafter referred to as *Acr-*GFP) mice were generated by mating *Acr*-GFP and *H3.3*-mCherry lines (both originally on a C57BL/6 background) [11,12]. In this line, green fluorescent protein (GFP) is expressed beginning at the mid-pachytene stage and continues throughout the subsequent stages of spermatogenesis. The *H3.3*-mCherry allele encodes a histone H3.3–mCherry fusion protein expressed under the control of the *Protamine 1* promoter. This fusion protein is normally expressed in elongating spermatids (steps 11–16). However, since the present study focused on meiotic progression, and the 2-D culture conditions did not support differentiation into round spermatids, mCherry fluorescence was not evaluated. Each line was maintained independently, with periodic backcrossing to ICR or C57BL/6, resulting in a mixed genetic background.

Testes were harvested from neonates aged 2.5–10.5 days *postpartum* (dpp). Mice were housed under specific-pathogen-free conditions (24 ± 1 °C, 55 ± 5% humidity, 14-hour light/10-hour dark cycle) and were fed commercial rodent chow (MF; Oriental Yeast) and acidified water (pH = 2.8–3.0) *ad libitum*. The latter is commonly used to suppress microbial contamination of drinking water systems. All animal experiments conformed to the Guide for the Care and Use of Laboratory Animals and were approved by the Institutional Committees of Laboratory Animal Experimentation at Yokohama City University (Protocol No. F-A-23–012).

### 2-D cell culture

**Fig 1:**
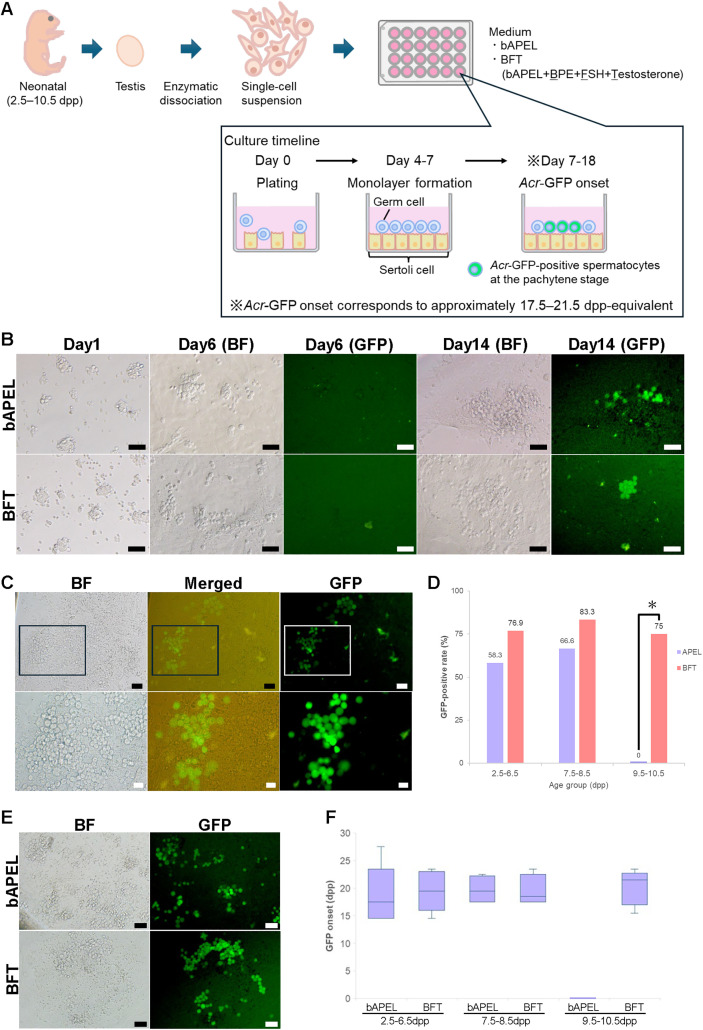
Expression of *Acr*-GFP under 2-D culture conditions. (A) Experimental design for cell culture of neonatal testes. Neonatal testes from *Acr*-GFP transgenic mice were enzymatically dissociated and cultured in 24-well plates to establish monolayer cultures. (B) Representative bright-field (BF) and GFP fluorescence images of testicular cell suspensions derived from *Acr*-GFP transgenic mice. Neonatal testes were harvested at 8.5 dpp and cultured in either bAPEL medium (top row) or BFT medium (bottom row) for 1, 6, or 14 days. GFP-positive cells (far right) indicate germ cells expressing *Acr*-GFP. Scale bars: 50 μm. (C) Representative fluorescence microscopy images of testicular cell suspensions derived from *Acr*-GFP transgenic testes, which were dissected at 9.5 dpp and cultured for 42 days in BFT medium. BF (left), Merged (center), and GFP fluorescence (right) images are shown. Scale bars: upper, 50 μm; lower, 20 μm. (D) Bar graph showing the percentage of wells with *Acr*-GFP-positive cells after culture in bAPEL or BFT medium, using testicular cells from 2.5–6.5 or 7.5–8.5, or 9.5–10.5 dpp mice. In the 2.5–6.5 dpp group, the GFP-positive rates were 58.3% (7/12) in bAPEL and 76.9% (10/13) in BFT (Fisher’s exact test, *P* = 0.41). In the 7.5–8.5 dpp group, the rates were 66.6% (4/6) in bAPEL and 83.3% (5/6) in BFT (*P* = 1.00). In the 9.5–10.5 dpp group, GFP-positive wells were detected only under BFT (6/8, *P* = 0.021), whereas no wells were positive in bAPEL (0/8). A well containing ≥1 *Acr*-GFP–expressing cell was defined as GFP-positive. Fisher’s exact test was used to compare the proportions of GFP-positive wells between bAPEL and BFT. *P < 0.05 (Fisher’s exact test) for the comparison between bAPEL and BFT in the 9.5–10.5 dpp group.(E) Representative fluorescence microscopy images of testicular cell suspensions derived from *Acr*-GFP transgenic testes, which were dissected at 2.5 dpp and cultured for 19 days in bAPEL and BFT medium. BF (left) and GFP fluorescence (right) images are shown. Scale bars: 50 μm. (F) Timing of GFP onset in cultures established from testes of different donor ages (2.5–6.5, 7.5–8.5, and 9.5–10.5 dpp) under bAPEL or BFT conditions. GFP onset is shown as the *in vivo*–equivalent age (dpp) at which *Acr*-GFP fluorescence first became detectable. Each box-and-whisker plot represents one group of biologically independent cultures; crosses indicate group means. The median GFP onset across individual groups ranged from 17.5 to 21.5 dpp. Sample sizes were as follows: 2.5–6.5 dpp: bAPEL (n = 7), BFT (n = 9); 7.5–8.5 dpp: bAPEL (n = 4), BFT (n = 5); 9.5–10.5 dpp: bAPEL (n = 8), BFT (n = 6)..

The experimental procedure is schematically illustrated in Fig 1A. Primary testicular cells were enzymatically dissociated from *Acr*-GFP mouse testes and seeded into 24-well plates. For tissue digestion, 5–6 testes were decapsulated and incubated in 3 mL of a 1 mg·mL ⁻ ¹ solution of Type IV collagenase derived from Clostridium histolyticum (Collagenase, Clostridiopeptidase A; Sigma-Aldrich^®^, St. Louis, MO, USA; Cat# C5138, CAS 9001-12-1), prepared in phosphate-buffered saline (PBS). Incubation was performed in a shaking water bath at 37 °C and 125 rpm for up to 15 minutes. After centrifugation (190 × g, 5 min, room temperature), the pellet was resuspended in 3 mL 0.05% trypsin (2.5% Trypsin, 10X; Gibco™, Thermo Fisher Scientific™, Waltham, MA, USA; Cat# 15090–046), gently pipetted, and incubated in a shaking water bath at 37 °C and 125 rpm for up to 10 minutes. Digestion was quenched with 3 mL DMEM + 10% fetal bovine serum (FBS), and the suspension was passed through a 40 µm nylon strainer. Cells were washed once, counted, and resuspended in basal APEL (bAPEL) (StemCell™ Technologies, Vancouver, BC, Canada; Cat# ST-05270) medium. APEL is a chemically defined, serum-free basal medium designed to support a wide range of adherent primary and stem cell–derived cultures [13–18]. 3.7 × 10^5^ cells in 1 mL medium were seeded per well of a 24-well plate (Sumitomo Bakelite, Tokyo, Japan; Cat# MS-80240). Three culture conditions were evaluated. The first condition, referred to as AlbuMAX medium, consisted of α-minimum essential medium (αMEM; Gibco™, Thermo Fisher Scientific™, Waltham, MA, USA; Cat# 12000–022) supplemented with AlbuMAX (Gibco™, Thermo Fisher Scientific™, Waltham, MA, USA; Cat# 11020–021) at a final concentration of 40 mg·mL ⁻ ¹. The second condition was bAPEL medium, which was composed of APEL supplemented with 1X Antibiotic-Antimycotic (Gibco™, Thermo Fisher Scientific™, Waltham, MA, USA; Cat# 15240062). The third condition, termed BFT medium, consisted of bAPEL further supplemented with bovine pituitary extract (BPE) (Takara Bio Inc., Kusatsu, Shiga, Japan; Cat# D13002; 50 µg·mL ⁻ ¹), follicle-stimulating hormone (FSH) (Sigma-Aldrich^®^, St. Louis, MO, USA; Cat# F4021-10UG; 200 ng·mL ⁻ ¹), and testosterone (FUJIFILM Wako Pure Chemical Corporation, Osaka, Japan; Cat# 208–08341; 10 µM). Serum-free conditions were employed to avoid the undefined and bioactive components present in fetal bovine serum, which can affect germ–somatic cell interactions and compromise the reproducibility of primary testicular cultures. The use of a chemically defined basal medium (APEL) minimized these potential confounding effects. Testes were obtained from three age groups to reflect the developmental transitions relevant to meiotic initiation under 2-D culture conditionss: 2.5–6.5 dpp, 7.5–8.5 dpp, and 9.5–10.5 dpp. These ranges reflect biologically distinct developmental stages rather than equivalent ages. Testes from 2.5–6.5 dpp contain only premeiotic germ cells and therefore initiate meiosis exclusively during culture, whereas testes from 7.5–8.5 dpp have already begun meiotic entry *in vivo* before dissociation [19]. Testes from 9.5–10.5 dpp were handled as a separate group because, in our preliminary experiments, they did not reach the pachytene stage in basal APEL medium and required hormonal supplementation (BFT) for meiotic progression. Grouping by developmental stage rather than exact daily age also ensured a feasible and reproducible experimental design across two media conditions (APEL and BFT) and multiple biological replicates. Cultures were maintained at 34 °C in an atmosphere of 5% CO₂ and 20% O₂, with medium changes performed every 3–4 days.

### Organ culture with PDMS ceiling chips

Detailed methodologies for this organ culture system have been described in previous publications [20–21]. After decapsulation, the testis parenchyma was cut into several appropriately sized fragments using forceps. The tissue fragments were placed on 1.5% agarose gel blocks that were half-soaked in culture medium, positioned in each well of a 12-well plate (CELLSTAR^®^,Greiner Bio-One, Kremsmünster, Austria; Cat# 665180). A PDMS ceiling chip (PC chip) was gently placed over each tissue fragment to promote 2-D spreading. The culture medium volume was adjusted to approximately half the height of the agarose gel (approximately 0.5 mL per well). The tissues were maintained in an incubator at 34 °C in an atmosphere of 5% CO₂ and 15% O₂. The culture medium, consisting of αMEM supplemented with AlbuMAX at a final concentration of 40 mg·mL ⁻ ¹, was replaced once a week.

### Observations

Cell cultures were examined at least once per week using an inverted fluorescence microscope (IX73, Olympus Corporation, Tokyo, Japan). To minimize environmental exposure and reduce observation time, not all wells were subjected to analysis. Instead, for each culture condition, a representative well was selected based on overall cell density, proliferative appearance, and, when applicable, the presence and distribution of GFP-positive cells. GFP-positive cells were then counted across the entire selected well using a 20 × objective lens to estimate their relative abundance.

Organ cultures destined for nuclear spread analysis were monitored in parallel using a fluorescence stereomicroscope (M205 FA, Leica Microsystems, Wetzlar, Germany).

### Definition of replicates and handling of wells

In this study, “n” denotes biologically independent experiments, each initiated from a separate preparation of testicular cell suspension. When testes from neonates of the same age were combined and dissociated together, the resulting suspension was counted a single experimental unit, regardless of the number of wells generated. Wells derived from the same dissociation were not regarded as biologically independent and were therefore not counted as separate replicates. Based on our experience, a single testis from a mouse at 2.5–6.5 dpp typically yielded enough cells to seed 1–2 wells suitable for culture, whereas a single testis from a mouse at 7.5–10.5 dpp yielded enough for 2–3 wells.

### Immunocytochemistry

Culture plate-based immunostaining was carried out as follows. The culture medium was aspirated, and each well was rinsed once with 1 mL PBS. Cells were fixed by adding 300 µL of 2% paraformaldehyde in PBS for 5 min at room temperature (RT). After fixation, the wells were washed three times with 500 µL PBS, followed by a 10 min incubation after the final wash. Subsequently, the wells were washed three times with PBS containing 0.2% Triton X-100 (PBST) for 10 minutes each. Non-specific binding sites were blocked with 5% bovine serum albumin (BSA) in PBST for 30 min at RT.

Primary antibodies (diluted in 5% BSA/PBST; see list below) were applied overnight at 4 °C. The next day, the wells were washed three times with 500 µL PBS, with a 10 min incubation after the final wash and incubated for 60 min at RT in the dark with species-specific Alexa Fluor-conjugated secondary antibodies (diluted 1:200 in 5% BSA/PBST). Nuclei were counterstained with Hoechst 33342 (Dojindo Laboratories, Kumamoto, Japan; Cat# 346–07951), diluted 1: 500 in PBS to a final concentration of 2 µg·mL ⁻ ¹ (200 µL per well, 10 min), followed by three additional PBS washes (500 µL each). Samples were stored in PBS and imaged with an IX73 inverted fluorescence microscope (Olympus).

Primary antibodies (dilution): rabbit anti SOX9 (Trans Genic Inc, KO608, 1: 100); rat anti-GCNA1[TRA98] (Abcam, ab82527, 1: 500); chicken anti-GFP (Abcam, ab13970, 1: 500); mouse anti-SCP3 (Abcam, ab97672, 1: 200); goat anti-GFRα1 (R&D Systems, AF560, 1: 200); mouse anti-PLZF (Active Motif, 39988, 1: 500).

Secondary antibodies (dilution 1: 200): Alexa Fluor 647 goat anti-rat (Invitrogen, A21247); Alexa Fluor 488 goat anti-chicken (Invitrogen, A11039); Alexa Fluor 647 goat anti-mouse (Invitrogen, A21235); Alexa Fluor 555 donkey anti-mouse (Invitrogen, A31570); Alexa Fluor 647 donkey anti-goat (Invitrogen, A21447) (see S1 Table).

### Nuclear spread of spermatocytes

Spermatocyte nuclear spreads were prepared based on the dry-down technique with modifications [22–24]. To evaluate meiotic prophase at the chromosomal level, SCP3, SCP1, and γ-H2A.X were used as markers of axial element formation, synapsis, and programmed DNA double-strand break dynamics, respectively. SCP3 localizes along chromosome axes, whereas SCP1 is restricted to synapsed regions of the synaptonemal complex [25,26]. γ-H2A.X marks programmed meiotic double-strand breaks and their resolution during prophase I [27]. For comparative analysis, nuclear spreads were prepared in parallel from 2-D cultures, organ-cultured testes, and *in vivo* testes at the corresponding developmental stage. Cells were collected from 2-D cell cultures, organ-cultured testis explants, or freshly isolated juvenile testes. For monolayer cultures, adherent cells were rinsed with PBS and detached using 0.25% trypsin at 37 °C for 10 min, followed by gentle trituration. Organ-cultured tissue was finely minced under a stereomicroscope with tungsten needles, digested with 1% collagenase for 15 min, centrifuged, and further dissociated using 0.25% trypsin for 10 min. Fresh testicular tissue was minced with scissors and digested in 0.25% trypsin under the same conditions. In all cases, trypsinization was terminated by adding DMEM containing 10% FBS, and the cell suspension was filtered twice through a 40 μm nylon mesh. Cells were pelleted (300 × g, 4 °C, 3 min) and washed three times with DMEM + 10% FBS.

The resulting pellets were resuspended in 100 μL PBS and mixed 1: 1 with hypotonic buffer containing 30 mM Tris, 50 mM sucrose, 17 mM trisodium citrate, 5 mM EDTA, and 0.5 mM dithiothreitol. After incubation for 8 min at RT, 800 μL PBS was added, and cells were gently mixed and collected by centrifugation.

Approximately 6.4 × 10⁴ cells were resuspended in PBS and mixed with 100 mM sucrose (pH = 8.2). A 30 μL aliquot of this mixture was dropped onto clean glass slides. An equal volume of 1% paraformaldehyde containing 0.1% Triton X-100 in 10 mM sodium borate (pH = 9.2) was added, and the suspension was evenly spread by slow pipetting. Slides were incubated in a dark, humidified chamber for 1 hour at RT, air-dried for 30 min, rinsed briefly with 0.4% DRIWEL, and fully dried before storage at −80 °C.

For immunofluorescence, slides were permeabilized with 0.5% Triton X-100 in PBS for 10 min, washed, and blocked in PBS containing 10% FBS and 2% donkey serum for 1 hour. Primary antibodies were applied overnight at 4 °C in the same blocking solution. Slides were then washed with PBS containing 0.05% Triton X-100 and incubated for 2 hours at RT with Alexa Fluor-conjugated secondary antibodies diluted in blocking buffer containing Hoechst 33342. After final washes, the samples were mounted with antifade reagent and imaged using an FV1000-MPE confocal microscope (Olympus). Primary antibodies were mouse anti-SCP3 (Abcam, ab97672, 1: 200), rabbit anti-γ-H2A.X (Abcam, ab81299, 1: 5,000), and rabbit anti-SCP1 (Abcam, ab175191, 1: 200). Secondary antibodies (1: 200 dilution) were species-specific Alexa Fluor 488 or 555 conjugates (Thermo Fisher Scientific) (see S1 Table).

### Statistical analysis

The GFP-positive rate was defined as the proportion of culture wells containing at least one *Acr*-GFP-expressing cell relative to the total number of wells analyzed under each condition. All statistical analyses were performed using EZR (version 1.68; Jichi Medical University, Japan). Fisher’s exact test was used to compare the proportions of GFP-positive wells between culture conditions [28]. The onset timing of GFP expression was compared using the two-tailed Mann–Whitney U test. A p-value < 0.05 was considered statistically significant. Box plots indicate the median, interquartile range (25th–75th percentile), and full range. Sample sizes are indicated in each figure legend.

## Results

### Effects of APEL medium and hormonal supplementation

To enable real time visualization of meiotic progression, we employed *Acr*-GFP transgenic mice, in which GFP is expressed under the control of the *Acrosin promoter*. In this line, GFP becomes detectable from the pachytene stage of meiotic prophase I and persists through the round and elongating spermatid stages [29]. We first tested AlbuMAX medium, which is commonly used in organ culture [3], to assess its ability to support meiosis in a 2-D cell culture system. In cultures derived from 7.5–8.5 dpp testes, somatic cells proliferated and formed a monolayer, but germ cell proliferation was not observed, and no GFP-positive cells were detected. We next tested bAPEL medium for its ability to support meiotic progression. In cultures derived from 7.5 to 8.5 dpp testes, GFP-positive cells first appeared under bAPEL conditions, where somatic cells often formed a confluent monolayer that supported germ cell proliferation and subsequent GFP expression (Fig 1B). In contrast, no GFP-positive cells were detected in any cultures derived from 9.5–10.5 dpp testes. This failure at later ages was unexpected, as these testes already contain spermatocytes *in vivo*, but the cultured cells did not show GFP expression under these conditions. To improve the somatic environment and facilitate meiotic progression, we supplemented bAPEL medium with BPE, FSH, and testosterone (BFT medium). BPE contains a variety of hormones, cytokines, mitogens, and growth factors that support cell proliferation and protect against oxidative stress [30]. FSH is known to stimulate Sertoli cell proliferation, and both FSH and testosterone have been shown to facilitate meiotic progression in mice [31]. Under BFT conditions, cells proliferated and formed a confluent monolayer within approximately one week. GFP-positive cells were subsequently detected around day 14, corresponding to an *in vitro* age of approximately 20 dpp (Fig 1B, 1C). Testicular cells from 7.5–8.5 dpp neonates were cultured in parallel in bAPEL and BFT conditions. The proportion of GFP-positive wells was 66.6% in bAPEL and 83.3% in BFT, but this difference was not statistically significant (Fisher’s exact test, *P* = 1.00; Fig 1D). In 9.5–10.5 dpp cultures, GFP-positive cells were detected only under BFT conditions, indicating that hormonal supplementation is required to support meiotic progression in more developmentally advanced testicular cells. These findings raised the question of whether the 2-D system can also support the initiation of meiosis from less mature testicular cells, which lack preexisting spermatocytes at the time of culture initiation.

To address this question, we next tested whether meiosis could be induced *de novo* from 2.5–6.5 dpp testes, which normally lack spermatocytes at the time of isolation. In both bAPEL and BFT media, cells adhered to the culture surface and expanded into monolayers, and GFP-positive cells subsequently emerged over time (Fig 1E). The proportions of GFP-positive wells were 58.3% in bAPEL and 76.9% in BFT, and this difference was not statistically significant (Fisher’s exact test, *P* = 0.41; Fig 1D). GFP expression in this system reflects developmental age rather than culture duration. *Acr*-GFP positive cells first appeared at approximately 17.5 to 21.5 dpp equivalent in both media (Fig 1F). The timing of GFP onset did not differ significantly across donor ages or media (Mann–Whitney U test, *P* = 0.661; Kruskal–Wallis test, *P* = 0.765).

### Sertoli cell organization and maintenance of undifferentiated spermatogonia

**Fig 2:**
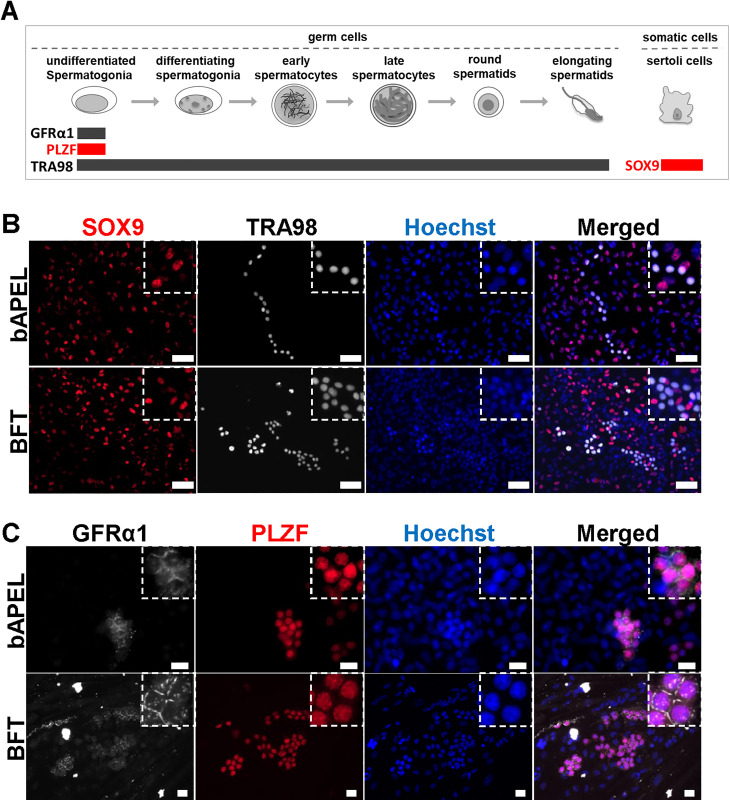
Immunocytochemistry of cells induced to undergo spermatogenesis in a 2-D culture system. (A) Schematic representation of mouse spermatogenesis and the expression of marker proteins used in this study. (B) Co-immunostaining for SOX9 (Sertoli cell marker, red) and TRA98 (germ cell marker, white) in cultures derived from 5.5 dpp Acr-GFP transgenic mice at day 22 of culture (corresponding to 27.5 dpp). SOX9-positive Sertoli cells and TRA98-positive germ cells were observed. Scale bar: 50 μm. (C) Co-immunostaining for GFRα1 (a marker of undifferentiated spermatogonia, white) and PLZF (a nuclear transcription factor expressed in undifferentiated spermatogonia, red) in cultures derived from 5.5 dpp Acr-GFP transgenic mice at day 22 of culture (equivalent to 27.5 dpp). Undifferentiated spermatogonia were preserved in the culture at day 22. Scale bar: 20 μm.

To identify the major testicular cell types present in culture, we used four well-established markers (Fig 2A). SOX9 is a nuclear transcription factor expressed specifically in Sertoli cells [32], whereas TRA98 recognizes GCNA1, a nuclear antigen broadly expressed in germ cells [33,34]. GFRα1 is a surface marker of undifferentiated spermatogonia [35]. PLZF is a transcription factor essential for maintaining the undifferentiated state [36]. SOX9-positive Sertoli cells expanded across the culture surface, forming a basal layer on which TRA98-positive germ cells were distributed (Fig 2B). After confirming the coexistence of Sertoli cells and germ cells, we next investigated whether the germ cell population included undifferentiated spermatogonia. Both GFRα1 and PLZF were detected on day 22 (Fig 2C), indicating that a pool of undifferentiated cells was maintained in culture. To assess the presence of steroid producing Leydig cells, immunostaining for 3β- hydroxysteroid dehydrogenase (3β-HSD) was performed. However, no clearly positive cells were detected in the 2-D culture system.

### Meiotic progression and chromosomal synapsis

**Fig 3:**
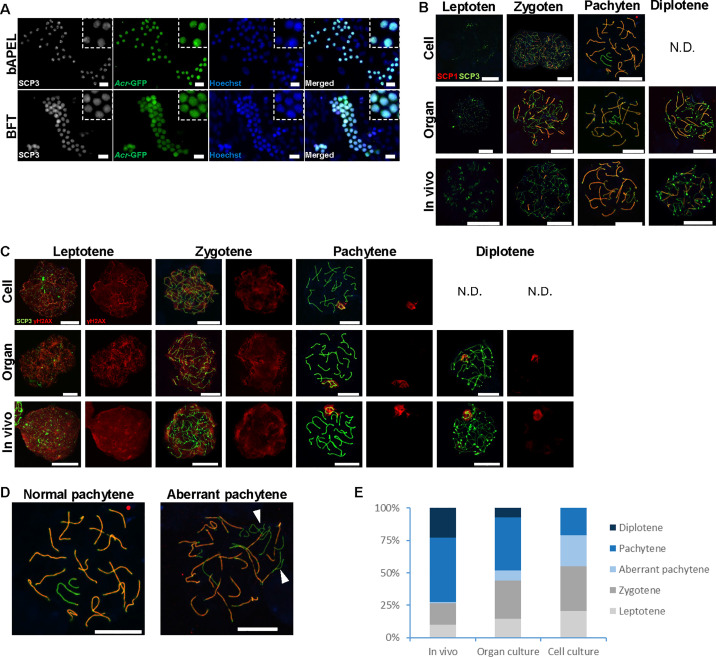
Comparison of meiotic prophase I progression in spermatocytes between cell culture, organ culture, and *in vivo* testes. (A) Co-immunostaining for SCP3 (white), an axial element protein of the synaptonemal complex, and GFP in cultures established from 5.5 dpp *Acr*-GFP transgenic mice and analyzed after 22 days of culture (corresponding to 27.5 dpp). GFP signal indicates *Acr*-GFP expression driven by the *Acr* promoter. All GFP-positive cells co-expressed SCP3, indicating their progression into meiotic prophase. Scale bar: 20 μm. (B) Representative chromatin spread images depicting leptotene, zygotene, pachytene, and diplotene stages of meiotic prophase I. Samples were obtained from 7.5 dpp *Acr*-GFP transgenic mouse testes after 21 days of *in vitro* cell culture (BFT medium) or *in vitro* organ culture (AlbuMAX medium), or directly from *in vivo* testes at 28 dpp. Cells were immunostained for SCP3 (green) and SCP1 (red), with DNA counterstained by Hoechst 33342 (blue). Scale bar: 10 μm. (C) Representative nuclear spread images depicting leptotene, zygotene, pachytene, and diplotene stages of meiotic prophase I. Samples were obtained from 7.5 dpp *Acr*-GFP transgenic mouse testes after 21 days of *in vitro* cell culture (BFT medium) or *in vitro* organ culture (AlbuMAX medium), or from *in vivo* testes at 28 dpp. Cells were immunostained for SCP3 (green) and γ-H2A.X (red), with DNA counterstained by Hoechst 33342 (blue). Scale bar: 10 μm. (D) Representative nuclear spread image of a pachytene-stage spermatocyte exhibiting synaptic abnormalities. The specimen was prepared from testicular cells isolated at 7.5 dpp and cultured for 21 days in BFT medium. Cells were immunostained for SCP3 (green) and SCP1 (red), with DNA counterstained by Hoechst 33342 (blue). Regions lacking SCP1 and stained only for SCP3 (arrowheads) indicate unsynapsed chromosome axes, suggestive of defective synapsis. Scale bar: 10 μm. (E) Proportion of spermatocytes at the leptotene, zygotene, aberrant pachytene, pachytene, and diplotene stages of meiotic prophase I in samples obtained from 7.5 dpp testes after 21 days of *in vitro* cell culture (BFT medium), 21 days of *in vitro* organ culture (AlbuMAX medium), or from *in vivo* testes at 28 dpp. Staging was determined based on SCP3 and SCP1 immunostaining following the criteria described in the Methods. The numbers of nuclei analyzed were n = 457 (*In vivo*), n = 365 (*In vitro* organ culture), and n = 418 (*In vitro* cell culture).

To confirm that GFP expression corresponded to genuine meiotic progression, we performed immunostaining for SCP3, an axial element of the synaptonemal complex [37]. All GFP-positive cells were also SCP3-positive, confirming that GFP expression marked cells in meiotic prophase (Fig 3A). To further characterize meiotic progression, we next analyzed nuclear spreads from 2-D cultures, organ cultures, and *in vivo* testes at the corresponding developmental stage. Cells were evaluated by double immunostaining for SCP3/SCP1 or SCP3/γ-H2A.X (Fig 3B–C). Cells were first evaluated by double immunostaining for SCP3 and SCP1 (Fig 3B). At the earliest stage, thin and discontinuous SCP3 lines were visible along chromosome cores and SCP1 was absent, a pattern diagnostic of leptotene. As synapsis progressed, SCP3 became continuous along the axes and SCP1 appeared on limited chromosomal segments, indicating zygotene. Cells showing full colocalization of SCP3 and SCP1 along the entire length of each bivalent were classified as pachytene. Cells in which SCP1 staining had disappeared while SCP3 persisted only as punctate foci at chromosome ends were classified as diplotene [24]. Diplotene-stage cells were absent in the 2-D cultures, indicating that meiotic progression under these conditions did not extend beyond pachytene. To assess programmed DNA double-strand break dynamics across meiotic prophase, we additionally performed double immunostaining for SCP3 and γ-H2A.X (Fig 3C). Meiotic stages were classified based on SCP3 morphology, using the same criteria described above. In leptotene and zygotene cells, γ-H2A.X signals were diffusely distributed throughout the nucleus, with stronger intensity in leptotene. In pachytene, γ-H2A.X signals became restricted to unsynapsed chromatin, with strong accumulation on the sex chromosomes, consistent with XY body formation. Upon entry into diplotene, γ-H2A.X signals on autosomes disappeared, and only faint residual staining persisted on the sex chromosomes. No diplotene-stage cells were detected in the 2-D cultures by SCP3/γH2AX staining, supporting the conclusion that meiotic progression under these conditions did not extend beyond pachytene. Since γ-H2A.X patterns alone are insufficient to reliably distinguish diplotene, classification was primarily based on SCP3 morphology. Using this classification, nuclear spread analysis confirmed progression to diplotene in both *in vivo* testes and organ cultures. In contrast, 2-D cultures advanced only to the pachytene stage, and several nuclei displayed aberrant chromosome synapsis (Fig 3D). Hereafter, we classified pachytene spermatocytes as aberrant pachytene if they exhibited at least one fully synapsed autosome together with at least one autosome synapsed along less than 50 percent of its length or completely unsynapsed. This criterion follows previous studies in which similar nuclei in mutant mice were designated as asynapsed pachytene [38,39]. Using this definition, aberrant pachytene spermatocytes were very rarely observed in *in vivo* testes, consistent with the low incidence quantified by nuclear spread analysis (0.7%). This percentage was calculated relative to all meiotic prophase I nuclei examined in each condition, with more than 300 nuclei evaluated per condition. In contrast, the proportion of aberrant pachytene nuclei among all analyzed cells was higher in organ culture (7.7%) and most pronounced in 2-D cell culture (23.7%), indicating an increased occurrence of synapsis defects in the 2-D cell culture condition. These findings indicate that the current 2-D system requires further optimization to support progression beyond the pachytene stage.

## Discussion

In this study, we developed a simplified 2-D culture system that maintains germ cell populations and supports meiotic progression up to the mid-pachytene stage under serum-free conditions. By combining a chemically defined basal medium with hormonal supplementation, this system offers a tractable platform for investigating early events of meiosis *in vitro*. Germ cells expressing *Acr*-GFP were identified as pachytene spermatocytes based on the combined expression patterns of SCP3, SCP1, and γ-H2A.X observed by immunostaining and nuclear spread analysis. However, no progression to the diplotene stage was observed, indicating an arrest at mid-pachytene.

Hormonal supplementation with BPE, FSH, and testosterone (BFT medium) improved somatic support and meiotic outcomes. Specifically, BFT medium promoted the formation of confluent SOX9-positive Sertoli cell layers and increased the frequency and persistence of GFP-positive cells. In particular, cultures derived from 9.5–10.5 dpp testes, which failed to generate GFP-positive cells in bAPEL, exhibited restored meiotic induction with BFT. At this developmental stage, Sertoli cells have reached a more mature state and exhibit limited proliferative activity. Although maturation enhances their capacity to support germ cells, their reduced proliferation hampers the formation of confluent monolayers *in vitro*, which may lead to insufficient somatic support and impaired meiotic induction [40]. Here, it should be noted that the absence of GFP-positive cells in 9.5–10.5 dpp cultures under bAPEL likely reflects insufficient reconstruction of the Sertoli-cell niche rather than an intrinsic inability of the medium to support differentiating germ cells. This interpretation is consistent with the fact that both bAPEL and BFT supported *de novo* meiotic induction when cultures were established from younger testes. Exogenous FSH likely reactivated the residual proliferative potential of Sertoli cells, as neonatal FSH treatment has been shown to increase Sertoli cell numbers and enhance subsequent spermatogenesis [31]. The prolonged presence of GFP-positive spermatocytes in BFT cultures further suggests enhanced survival and reduced apoptosis under hormonally enriched conditions.

The timing of GFP onset remained broadly consistent across donor ages and media conditions, with a median onset ranging from 17.5 to 21.5 dpp in most cultures. Nonetheless, when compared to organ culture, the onset of GFP expression in our 2-D system was slightly delayed but consistently reproducible. Previous studies using the same *Acr*-GFP transgenic line have shown that, in organ culture, GFP-positive pachytene spermatocytes first appear at around 14.5 dpp, closely matching the *in vivo* schedule of meiotic onset [41]. Thus, the onset of GFP expression in our 2-D system was modestly but consistently delayed relative to both organ culture and *in vivo* conditions. This delay likely reflects two biological mechanisms. First, enzymatic dissociation primarily affects germ cells, eliminating those already undergoing or primed for meiosis and thereby resetting the germ cell pool to a premeiotic state [42]. This interpretation is consistent with our observation that GFP-positive spermatocytes present *in vivo* were seldom retained after plating, whereas undifferentiated spermatogonia survived and later initiated meiosis *de novo*. Second, tissue dissociation mainly impacts somatic cells, particularly Sertoli cells, by disrupting the native architecture, such that Sertoli cells require time to re-establish the supportive environment necessary to initiate meiosis [43]. In this context, a moderate delay does not indicate dysfunction but rather reflects the reasonable requirements for both resetting the germ cell pool to a premeiotic state and somatic reorganization after tissue disaggregation.

In addition, several nuclei from 2-D cultures exhibited aberrant synapsis, including unsynapsed SCP3-positive stretches and fragmented bivalents. These abnormalities may result from subtle variations in temperature, oxygen concentration, or hormonal composition, underscoring the need for further microenvironmental optimization. In contrast, both organ cultures and *in vivo* controls showed normal synapsis and progression beyond pachytene, highlighting the current limitations of the 2-D system. Compared with organ culture, the 2-D approach provides several practical advantages, including uniform exposure of dissociated cells to nutrients and hormones and a more controllable culture environment, particularly when defined media such as APEL are used. The monolayer configuration also enhances optical accessibility, facilitating detailed morphological and temporal observation. However, the absence of three-dimensional architecture and organized somatic support in 2-D culture likely underlies its inability to support complete meiotic progression, whereas organ culture can reproduce spermatogenesis through to functional sperm. Thus, the two systems offer complementary strengths depending on the experimental purpose. In our previous studies, we developed a microfluidic device-based organ culture platform that enabled improved control over the local microenvironment, including oxygen and nutrient gradients, while preserving tissue architecture. This approach allowed for enhanced observation and manipulation compared to organ culture and may serve as a bridge between traditional 3-D systems and simplified 2-D cultures [44,45].

Compared to previous studies, our system demonstrated more gradual meiotic progression than *in vivo*, yet avoided the premature acceleration observed in some *in vitro* models. One previous study reported *in vitro* completion of meiosis in just 14 days by co-culturing embryonic stem cell-derived primordial germ cell-like cells with neonatal testicular somatic cells [10]. However, this accelerated timeline deviates substantially from *in vivo* kinetics and may raise concerns regarding the accuracy of developmental recapitulation. Similarly, other 2-D co-culture systems using spermatogonial stem cells and immortalized Sertoli cells have been reported to induce meiosis but were accompanied by numerous aberrant M-phase-like cells and incomplete synapsis [46]. In contrast, our 2-D culture system supported meiotic progression with a modest delay, recapitulating early prophase I events more faithfully and without producing the flower-shaped M-phase-like chromosome aberrations described in that study [46] or other abnormal synapsis patterns.

A defining advantage of our approach is its simplicity and reliance on bAPEL, a serum-free, chemically defined basal medium. Unlike previous models that require strong exogenous differentiation cues such as retinoic acid or BMPs, our system relies on bAPEL with moderate supplementation of BPE, FSH, and testosterone. While retinoic acid was not directly added, we cannot exclude the possibility that residual RA signals within the testicular cell population contributed to meiotic induction. Although BPE is an undefined supplement, its use in combination with a chemically defined basal medium keeps the overall formulation relatively tractable. In this study, systematic nuclear spread analyses were applied to the simplified 2-D culture system, allowing quantitative definition of pachytene-stage arrest and synapsis defects at the chromosomal level. This level of resolution complements marker-based assessments and helps clarify the limitations of meiotic progression in a 2-D culture environment. This simplified 2-D culture system provides an experimentally accessible platform for analyzing early meiotic progression *in vitro*.

## Supporting information

S1 Table List of primary and secondary antibodies used in immunocytochemistry and nuclear spread analyses. (DOCX)

S1 Raw data GFP Positive Rate underlying Fig 1D. (XLSX)

S2 Raw data GFP onset timing data underlying Fig 1F. (XLSX)

S3 Raw data Nucleus-level meiotic prophase I staging data underlying Fig 3E. (XLSX)
