# Supplementary material for: A simplified two-dimensional culture system supports meiotic progression during mouse spermatogenesis in vitro
Source: PLoS One. 2026 Feb 5;21(2):e0342007. doi: 10.1371/journal.pone.0342007 (PMC12875477; doi:10.1371/journal.pone.0342007)
Supplement: S1 Table — (DOCX) [file pone.0342007.s001.docx]

**S1 Table. List of primary and secondary antibodies used in immunocytochemistry and nuclear spread analyses.**
This table summarizes all antibodies used in the present study, including their host species, providers, catalog numbers, batch numbers, and working dilutions. Primary antibodies were used to detect germ cell, Sertoli cell, or meiotic markers (SOX9, TRA98/GCNA1, GFP, SCP3, γ-H2A.X, SCP1, GFRα1, PLZF). Secondary antibodies were species-specific Alexa Fluor–conjugated antibodies used at the indicated dilutions.

| Antibody | Clone | Provider | Catalog number | Batch Number | Dilution |
| --- | --- | --- | --- | --- | --- |
| Primary antibody |  |  |  |  |  |
| anti mouse Sox9 polyclonal antibody | n/a | Trans Genic Inc., Kobe, Japan | KO608 | TG180823 | 1 : 100 |
| anti GCNA1 rat monoclonal antibody [TRA98] | TRA98 | Abcam^®^, Cambridge, UK | ab82527 | 1098726-6 | 1 : 500 |
| anti GFP chicken polyclonal antibody | n/a | Abcam^®^, Cambridge, UK | ab13970 | GR3361051-13 | 1 : 500 |
| anti SCP3 mouse monoclonal antibody | Cor 10G11/7 | Abcam^®^, Cambridge, UK | ab97672 | 1009233-36 | 1 : 200 |
| anti gamma H2A.X rabbit monoclonal antibody | EP854(2)Y | Abcam^®^, Cambridge, UK | ab81299 | 1094108-13 | 1 : 5000 |
| anti SCP1 rabbit monoclonal antibody | EP7918 | Abcam^®^, Cambridge, UK | ab175191 | 1059349-4 | 1 : 200 |
| anti GFRα1 goat polyclonal antibody | n/a | R&D Systems^®^, Minneapolis, MN, USA | AF560 | BQE0524011 | 1 : 200 |
| anti PLZF mouse monoclonal antibody | 2A9 | Active Motif^®^, Carlsbad, CA, USA | 39988 | 23221138-11 | 1 : 500 |
| Secondary antibody |  |  |  |  |  |
| Alexa Fluor 555 donkey anti-rabbit antibody | n/a | Thermo Fisher Scientific^TM^, Waltham, MA, USA | A32794 | YH376319 | 1 : 200 |
| Alexa Fluor 647 goat anti-rat antibody | n/a | Thermo Fisher Scientific^TM^, Waltham, MA, USA | A21247 | 2633536 | 1 : 200 |
| Alexa Fluor 488 goat anti-chicken antibody | n/a | Thermo Fisher Scientific^TM^, Waltham, MA, USA | A11039 | 2566343 | 1 : 200 |
| Alexa Fluor 647 goat anti-mouse antibody | n/a | Thermo Fisher Scientific^TM^, Waltham, MA, USA | A21235 | 1922319 | 1 : 200 |
| Alexa Fluor 555 donkey anti-mouse antibody | n/a | Thermo Fisher Scientific^TM^, Waltham, MA, USA | A31570 | 2831375 | 1 : 200 |
| Alexa Fluor 647 donkey anti-goat antibody | n/a | Thermo Fisher Scientific^TM^, Waltham, MA, USA | A21447 | 774898 | 1 : 200 |
